# The mediating role of social support and resilience between self-efficacy and prenatal stress: a mediational analysis

**DOI:** 10.1186/s12884-023-06184-2

**Published:** 2023-12-16

**Authors:** Yanchi Wang, Jian Gu, Feng Zhang, Xujuan Xu

**Affiliations:** 1 Affiliated Nantong Hospital of Shanghai University (The Sixth People’s Hospital of Nantong), Nantong, Jiangsu China; 2 Medical School of Nantong University, Nantong, Jiangsu China; 3 Department of Nursing, Affiliated Hospital of Nantong University, Nantong, 226001 Jiangsu China; 4 Department of Epidemiology and Medical Statistics, School of Public Health, Nantong University, Nantong, Jiangsu China; 5 Medical School (School of Nursing), Nantong University, Nantong, 226001 Jiangsu China

**Keywords:** Prenatal stress, Self-efficacy, Resilience, Social Support, Mediating effect

## Abstract

**Background:**

Prenatal stress is a highly prevalent mental disorder experienced by pregnant women. This study assessed the prevalence and influencing factors of prenatal stress and investigated the mediating role of social support and resilience between self-efficacy and prenatal stress among pregnant women in China.

**Methods:**

A convenience sample comprising 1071 pregnant women from three hospitals in Nantong, Jiangsu Province, China, was recruited between February and June 2023. These participants completed a set of general survey questionnaires and were assessed using the Pregnancy Pressure Scale, Perceived Social Support Scale, the 10-item Connor–Davidson Resilience Scale, and the Chinese version of the General Self-Efficacy Scale. Furthermore, a hierarchical multiple regression model was employed to investigate the relevant factors and mediators of prenatal stress symptoms. A structural equation model was used to examine the mediating role of social support and resilience in the relationship between self-efficacy and prenatal stress.

**Results:**

The results of the multivariate regression analysis indicated significant associations between prenatal stress and parity, self-efficacy, social support, and resilience (*P* < 0.001). Self-efficacy accounted for 35.33% of the total effect, with a direct effect of -2.5306 (95% confidence interval [CI]: -4.0309 to -1,0303). Further examination through mediation analysis revealed the mediating roles of social support and resilience in the relationship between self-efficacy and prenatal stress. The mediating effect of social support was − 1.5933 (95% CI: -2.2907 to -0.9496), accounting for 22.24% of the total effect. Similarly, resilience exhibited a mediating effect of -3.0388 (95% CI: -4.3844 to -1.7135), accounting for 42.43% of the total effect.

**Conclusion:**

The mediation analysis revealed that among pregnant women in China, the influence of self-efficacy on prenatal stress is channelled through social support and resilience. Therefore, enhancing social support, resilience, and self-efficacy might alleviate prenatal stress.

## Introduction

Pregnancy is a period that often brings about significant stress for most women [1]. The confluence of hormonal and physiological alterations [2], coupled with exposure to stressful events during pregnancy, may exert detrimental effects on the physical and mental well-being of expectant mothers. Literature has demonstrated that three out of four pregnant women report experiencing symptoms indicative of stress [3]. The incidence of prenatal stress during pregnancy varies from 25% [4] to 75% [5]. One study reported the prevalence of prenatal stress in mainland China as 91.86% [6]. Pregnancy is linked to a multitude of physiological and psychological changes, as well as experiences of considerable stress [7]. Prenatal stress encompasses concerns related to relationships, the impending parental role, physical changes, the delivery process, as well as the health and future care of the infant [8]. Elevated stress levels during this crucial period are associated with an increased risk of mental disorders [9]. Women who experience higher levels of stress during pregnancy are more prone to adverse effects on both themselves and their infants [10]. Additionally, prenatal stress has been linked to adverse maternal and perinatal outcomes, including premature rupture of membranes, preterm labour, and the birth of small-for-gestational-age foetuses [11]. There is evidence of a connection between intrauterine stress and its potential repercussions on cognitive and motor development, as well as behavioural alterations in childhood [12]. The prevalence of psychological disturbances tends to rise in women during pregnancy and the postpartum period [13]. Research indicates that the peak of mental health problems during pregnancy occurs in the third trimester, and this trimester exerts a more significant impact on postpartum mood [14, 15]. Consequently, women in the third trimester require increased attention, appropriate care, and follow-up to ensure timely detection and intervention.

Numerous research studies have reported that self-efficacy is the significant determinant of critical structures and behaviours. The impact of self-efficacy on the physical and psychological state of mothers, enabling individuals to manage stress rationally and constructively [16]. Furthermore, individuals possessing high levels of self-efficacy demonstrate enhanced abilities to navigate challenges and cope with stressful events compared to those with lower self-efficacy levels. This relationship is underscored by research findings that highlight an inverse connection between maternal stress and women’s self-efficacy [17, 18]. Social support, defined as an individual’s perception of external assistance, exhibits a negative correlation with prenatal stress among pregnant women. In essence, higher levels of social support are associated with milder prenatal stress during pregnancy [19]. A comprehensive review has also corroborated the role of social support and self-efficacy in alleviating prenatal stress among women with gestational diabetes [20]. Together, social support and self-efficacy emerge as determinants of prenatal stress during pregnancy [21]. Higher levels of self-efficacy in pregnant women have the potential to enhance their ability to navigate and cope with stressful situations. Pregnant women with elevated self-efficacy tend to exhibit lower levels of stress and are more likely to benefit from stronger social relationships and support networks. On the other hand, women with lower self-efficacy levels often report a higher prevalence of negative experiences throughout their pregnancy [22]. Therefore, we hypothesize that pregnant women with high self-efficacy are more likely to receive greater social support, leading to a reduction in prenatal stress.

Resilience is another positive psychological asset for preventing mental disorders. It constitutes a dynamic process that enables individuals, regardless of their life stage, with the ability to confront adversity, recover from hardship, manage unpleasant emotions, and adapt to changes [23]. Resilience encompasses an array of personal resources that act as a shield, safeguarding individuals from the negative effects of stressors. In the context of maternal well-being, maternal resilience might play a protective role against maternal stress during pregnancy and its negative consequences [24]. Furthermore, self-efficacy enhances an individual’s capacity for resilience, potentially preventing mental health issues during pregnancy [25]. Additionally, a study indicated that resilience plays a mediating role between self-efficacy and prenatal anxiety symptoms among Chinese pregnant women [26]. Therefore, it is hypothesised that psychological resilience might also mediate the relationship between self-efficacy and prenatal stress symptoms.

**Fig. 1:**
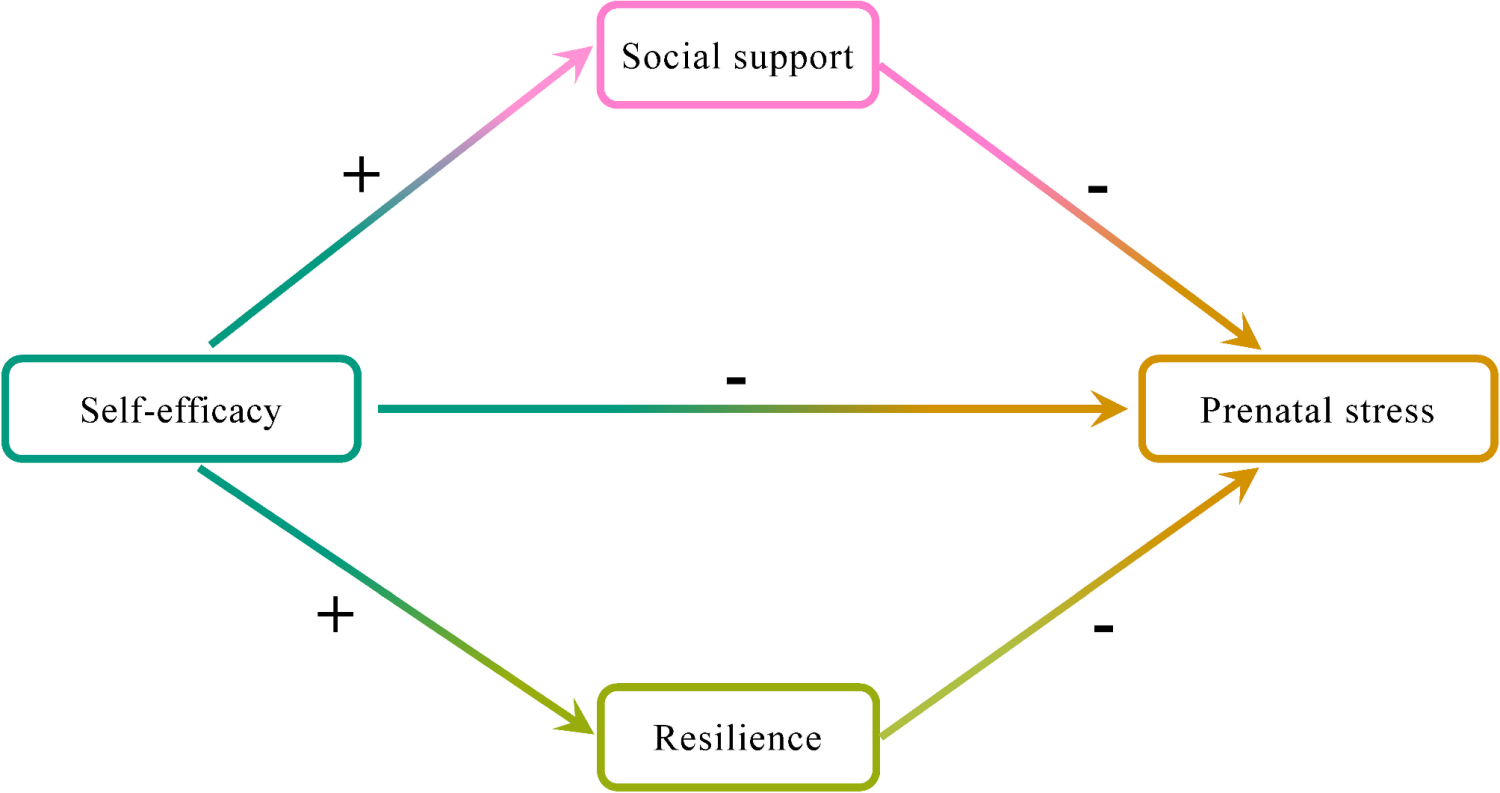
Hypothetical model of this study

According to previous studies, self-efficacy, social support, and resilience affect prenatal stress [27]. Moreover, self-efficacy affects social support; and resilience partially mediates the relationship between self-efficacy and the mental health of pregnant women. However, no study has examined the interconnected mediation effects among these three factors (self-efficacy, social support, and resilience) and prenatal stress. Therefore, based on the aforementioned literature and established theories, it was hypothesised that social support and resilience serve as parallel mediators for the association between self-efficacy and prenatal stress (Fig. 1, Hypothetical Model).

This study aimed to evaluate the prevalence of prenatal stress, identify influencing factors, and investigate how social support and resilience mediate the relationship between self-efficacy and prenatal stress. It is believed that the findings of this study will deepen our understanding of the mechanism underlying prenatal stress, which can help us develop effective prevention and interventional strategies in the future.

## Materials and methods

### Participants

A total of 1088 pregnant women were surveyed, of which 5 dropped out, 10 had incomplete data, and 2 provided invalid answers. Consequently, the final analysis included 1071 participants, yielding a response rate of 98.44%. The inclusion criteria were as follows: (a) maternal age ≥ 18 years; (b) ability to actively participate in the survey and engage in regular communication; and (c) ability to comprehend the content of the questionnaire and complete it independently. The exclusion criteria were as follows: (a) the presence of mental disorders, such as schizophrenia, severe depression, anxiety, mania, and bipolar affective disorder; (b) abnormal pregnancy, conditions, such as foetal malformation; and (c) a documented history of mental illness or cognitive dysfunction.

### Data collection

Using a convenience sampling approach, pregnant women from the Department of Obstetrics and Gynaecology in three tertiary hospitals in Nantong, Jiangsu Province, China, were selected as research participants. Primary data from these participants were obtained through face-to-face questionnaires administered between February and June 2023. At the onset of our interaction with the participants, who were attending antenatal clinics for foetal heart monitoring, the purpose and significance of the study were explained. Furthermore, it was conveyed that participation was entirely voluntary and that the participants could withdraw from the study at any point without any consequences. Informed consent was obtained from all participants prior to study commencement. The consent form outlined the study details, including its objectives, data collection procedures, potential risks and benefits, confidentiality measures, and contact information for any inquiries or concerns. The participants were informed that the questionnaire aimed to obtain information related to their postpartum experience, which would be kept anonymous and confidential. The ethics committee of the Affiliated Hospital of Nantong University (approval number: 2022-K50-01) approved this study.

### Measurement

#### Sociodemographic characteristics

Our research team members devised a questionnaire to collect information regarding the general characteristics of pregnant women. The primary variables encompassed the following: (1) fundamental demographic details, including age, place of residence, educational background of the pregnant woman, educational background of her husband, marital status (first marriage), and the family’s monthly income and (2) factors related to maternity, including abortion history, parity, pregnancy-related complications, assisted reproduction, gestational age, and underlying diseases.

#### Pregnancy pressure scale (PPS)

The PPS, originally developed by Chen et al. [28] in Taiwan, China, is a self-report assessment tool. Comprising 30 items, the PPS employs a 4-point scale (0 = not at all, 1 = mild, 2 = moderate, and 3 = severe). Its design is tailored to align with the Chinese cultural framework. The instrument assesses stress related to maternal and child health and safety, the recognition of parental roles, as well as changes in body shape and physical activity during pregnancy. Higher scores on the PPS indicate elevated levels of stress experienced during pregnancy. Prior research has established the instrument’s reliability among Chinese women [29]. In this study, the PPS was used to measure prenatal stress, achieving a Cronbach’s alpha coefficient of 0.94.

#### Perceived social support (PSS) scale (PSSS)

The Chinese version of the PSSS, developed by Zimet [30], is used to evaluate PSS. Comprising 12 items, this scale measures how individuals perceive social support from their families (four items), friends (four items), and significant others (four items). Additionally, these items can be analysed in terms of internal and external aspects of family support (intrafamily support and extrafamily support). Each item is scored on a 7-point Likert scale ranging from 1 (very strongly disagree) to 7 (very strongly agree), with higher scores indicating a greater perception of social support. The scale’s robust psychometric characteristics have been confirmed within the Chinese population [31]. The Cronbach’s alpha coefficient of the three domains was 0.88, and the test-retest reliability was 0.85.

#### 10-item Connor–Davidson Resilience Scale (CD-RISC-10)

The CD-RISC-10, a scale co-developed by Connor and Davidson [32], comprises 10 items. The Cronbach’s alpha coefficient for CD-RISC-10 was 0.85, indicating good reliability and construct validity. In this study, the Chinese version of CD-RISC-10 was used, which was translated and revised by Chinese scholars. The translated version exhibited a Cronbach’s alpha coefficient of 0.92, indicating its robust psychometric properties, including internal consistency, consequential validity, and criterion-related validity [33]. This scale was also administered to pregnant women in China [34]. Participants rated each of the 10 items on a 5-point Likert scale, with scores ranging from 0 to 4, corresponding to the responses: “never”, “seldom”, “sometimes”, “frequently”, and “always”, respectively. The CD-RISC-10 score was calculated as the sum of the scores for all items, with higher scores indicating greater resilience.

#### Chinese version of the general self-efficacy scale (GSES)

Self-efficacy was assessed using the Chinese version of GSES, which was developed by Schwarzer [35]. The scale has been previously used in Chinese populations, where it demonstrated good reliability and validity [36]. Comprising 10 items, it employs a 4-point Likert scale for scoring. A higher overall score indicates a higher degree of self-efficacy. In the present study, the Cronbach’s alpha coefficient for GSES was 0.953.

### Statistical analyses

The general characteristics were presented using descriptive analyses (such as mean, standard deviation [SD], frequency, and constituent ratio). Considering that the prenatal stress score data did not adhere to a normal distribution, the prenatal stress scores were normalised using rank-based inverse-normal transformation (INT) prior to conducting the statistical analyses. After INT, the prenatal stress scores conformed to the normal distribution. Student’s t-tests and one-way analysis of variance were used to assess the differences in prenatal stress scores (after INT) for each group’s demographic characteristics, while multiple linear regression analysis was used to assess the association between each scale and prenatal stress levels (prenatal stress scores after INT).

The correlation between variables was analysed using Pearson’s correlation analysis. Parallel mediation modelling analyses were used to investigate the association between social support, resilience, self-efficacy, and prenatal stress. The bootstrap method was applied with 5000 iterations to examine the mediating effects, generating 95% confidence intervals (CIs) for our findings. SPSS version 25.0 (IBM Corp., Armonk, NY) and SPSS PROCESS macro version 3.3 were used to perform statistical analyses. Variables used in multiple linear regression analysis and parallel mediation modelling analyses encompassed social support, resilience, self-efficacy, and prenatal stress scores. The random forest analysis, including variable importance and the SHAP summary plot were constructed using Python3.8. All figures were created using R version 3.6.2. Type I error was set at *P* < 0.05 (two-sided) for all statistical analyses.

## Results

### Characteristics of the study population

Data on general characteristics, including age, educational background of the pregnant woman, educational background of her husband, place of residence, family monthly income, abortion history, parity, assisted reproduction, pregnancy complications, gestational age, underlying diseases, and prenatal stress values were obtained (Table 1**)**. The prenatal stress score (mean ± SD) of the demographic characteristics of all groups were not statistically significant (*P > 0.05*), except for the age of the pregnant woman (*P = 0.010*), family monthly income (*P = 0.036*), and parity (*P < 0.001*).

**Table 1:** Characteristics of the subjects enrolled in this study

Variables		Scores of prenatal stress		*P*		Variables		Scores of prenatal stress		*P*
		N (%)	Mean ± SD					N (%)	Mean ± SD	
Age	< 30 years	554 (51.7)	0.43 ± 0.38	0.010		Gestational weeks	32–35^+ 6^	775 (72.4)	0.39 ± 0.35	0.087
	≥ 30 years	517 (48.3)	0.37 ± 0.35				36–40	296 (27.6)	0.44 ± 0.42	
Education (oneself)	Below bachelor	402 (37.5)	0.38 ± 0.41	0.076		Parity	Primipara	775 (72.4)	0.44 ± 0.37	< 0.001
	Bachelor or above	669 (62.5)	0.42 ± 0.34				Multipara	296 (27.6)	0.32 ± 0.35	
Education (husband)	Below bachelor	440 (41.1)	0.41 ± 0.40	0.671		Assisted reproduction	No	928 (86.6)	0.40 ± 0.37	0.730
	Bachelor or above	631(58.9)	0.40 ± 0.34				Yes	143 (13.4)	0.41 ± 0.37	
Family monthly income (CNY ¥)	< 5000	29 (2.7)	0.51 ± 0.47	0.036		Residence	Downtown	623 (58.2)	0.40 ± 0.36	0.981
	5000–10,000	352 (32.9)	0.41 ± 0.40				Town	273 (25.5)	0.40 ± 0.36	
	10,001–20,000	519 (48.5)	0.42 ± 0.35				Village	175 (16.3)	0.41 ± 0.41	
	> 20,000	171 (16.0)	0.34 ± 0.33			Complications of pregnancy	No	822 (76.8)	0.40 ± 0.36	0.934
First marriage	Yes	1018 (95.1)	0.41 ± 0.37	0.068			Yes	249 (23.2)	0.41 ± 0.38	
	No	53 (4.9)	0.31 ± 0.34			Working	Yes	419 (39.1)	0.42 ± 0.36	0.187
Underlying disease	Yes	58 (5.4)	0.42 ± 0.36	0.728			No	652 (60.9)	0.39 ± 0.37	
	No	1013 (94.6)	0.40 ± 0.37			Gestational hypertension	Yes	17(1.6%)	0.38 ± 0.35	0.770
Gestational diabetes	Yes	153(14.3%)	0.40 ± 0.41	0.993			NO	1054(98.4%)	0.40 ± 0.37	
	No	918(85.7%)	0.40 ± 0.36			Hypothyroidism in pregnancy	Yes	60(5.6%)	0.40 ± 0.33	0.945
Hyperthyroidism during pregnancy	Yes	15(0.4%)	0.52 ± 0.45	0.203			NO	1011(94.4%)	0.40 ± 0.37	
	No	1056(98.6%)	0.40 ± 0.37							

### Prenatal stress levels across the study sample

Of the 1071 participants, 128 pregnant women (11.951%) exhibited a prenatal stress score of 0 points, indicating an absence of prenatal stress. Mild prenatal stress, denoted by scores ranging from 0.001 to 1.000 points, was observed in 870 (81.233%) pregnant women. Additionally, 72 pregnant women (6.723%) demonstrated moderate prenatal stress, with scores ranging from 1.001 to 2.000 points. Only one pregnant woman (0.093%) exhibited severe prenatal stress, with a score ranging from 2.001 to 3.000. The prevalence of prenatal stress in China was 88.05%.

### Correlations between social support, resilience, self-efficacy, and prenatal stress

Increased levels of self-efficacy (β: -7.163, 95% CI: -8.146 to -6.179, *P* < 0.001), increased social support (β: -0.416, 95% CI: -0.478 to -0.355, *P* < 0.001), and greater resilience (β: -0.716, 95% CI: -0.806 to -0.626, *P* < 0.001) were significantly associated with decreased prenatal stress levels (Table 2).

**Table 2:** Results of univariate analysis of prenatal stress

Variables	β	SE	95% CI		*P*
			Lower	Upper	
Self-efficacy	-7.163	0.501	-8.146	-6.179	< 0.001
Social Support	-0.416	0.031	-0.478	-0.355	< 0.001
Resilience	-0.716	0.046	-0.806	-0.626	< 0.001

### Multivariate analysis of prenatal stress

**Fig. 2:**
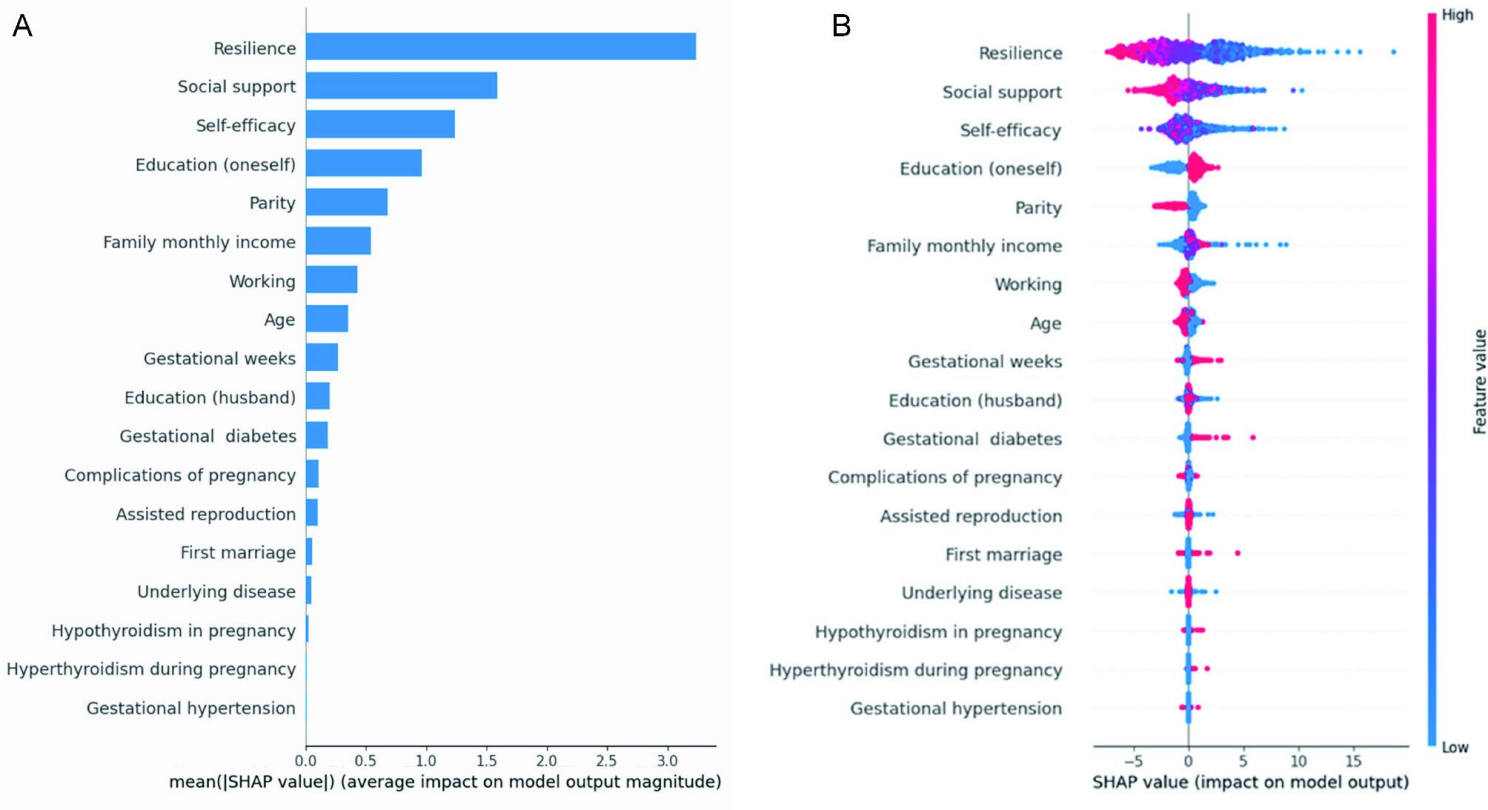
The results of the random forest analysis. **A**: variable importance; **B**: the SHAP summary plot

Multivariate logistic regression analysis found that increased self-efficacy (β: -2.586, 95% CI: -4.069 to -1.103, *P* < 0.001), increased social support (β: -0.210, 95% CI: -0.281 to -0.140, *P* < 0.001), greater resilience (β: -0.350, 95% CI: -0.495 to -0.205, *P* < 0.001), and parity (β: -3.370, 95% CI: -4.664 to -2.077, *P* < 0.001) were significantly associated with decreased prenatal stress levels (Table 3). In addition, the results of the random forest analysis, including variable importance and the SHAP summary plot were shown in Fig. 2.

**Table 3:** Results of multivariate analysis of prenatal stress

Variables	β	SE	95% CI		*P*
			Lower	Upper	
Self-efficacy	-2.586	0.756	-4.069	-1.103	< 0.001
Social Support	-0.210	0.036	-0.281	-0.140	< 0.001
Resilience	-0.350	0.074	-0.495	-0.205	< 0.001
Parity	-3.370	0.659	-4.664	-2.077	< 0.001

### Correlation analysis

As presented in Table 4, the average prenatal stress score was 0.368 ± 0.404. A significant and negative correlation was observed between prenatal stress and self-efficacy (r = -0.401, *P* < 0.001), social support (r = -0.376, *P* < 0.001), and resilience (r = -0.433, *P* < 0.001).

**Table 4:** Mean, standard deviation (SD), and correlations for study variables (N = 1071)

Variables	Prenatal Stress	Self-efficacy	Social Support	Resilience
Prenatal Stress	1			
Self-efficacy	-0.401^***^	1		
Social Support	-0.376^***^	0.485^***^	1	
Resilience	-0.433^***^	0.773^***^	0.562^***^	1
Mean	0.368	2.777	72.055	28.200
Standard deviation (SD)	0.404	0.617	9.964	6.666

***P < 0.001 (two-tailed test)

### Mediation analysis

The correlation analysis revealed a significant association between self-efficacy and prenatal stress, indicating that greater self-efficacy was associated with lower prenatal stress levels. However, upon incorporating mediators into the model, the direct effect of self-efficacy on prenatal stress was partially mediated, resulting in a coefficient of -2.5306 (95% CI: -4.0309 to -1.0303, *P* < 0.001), accounting for only 35.33% of the total effect.

**Fig. 3:**
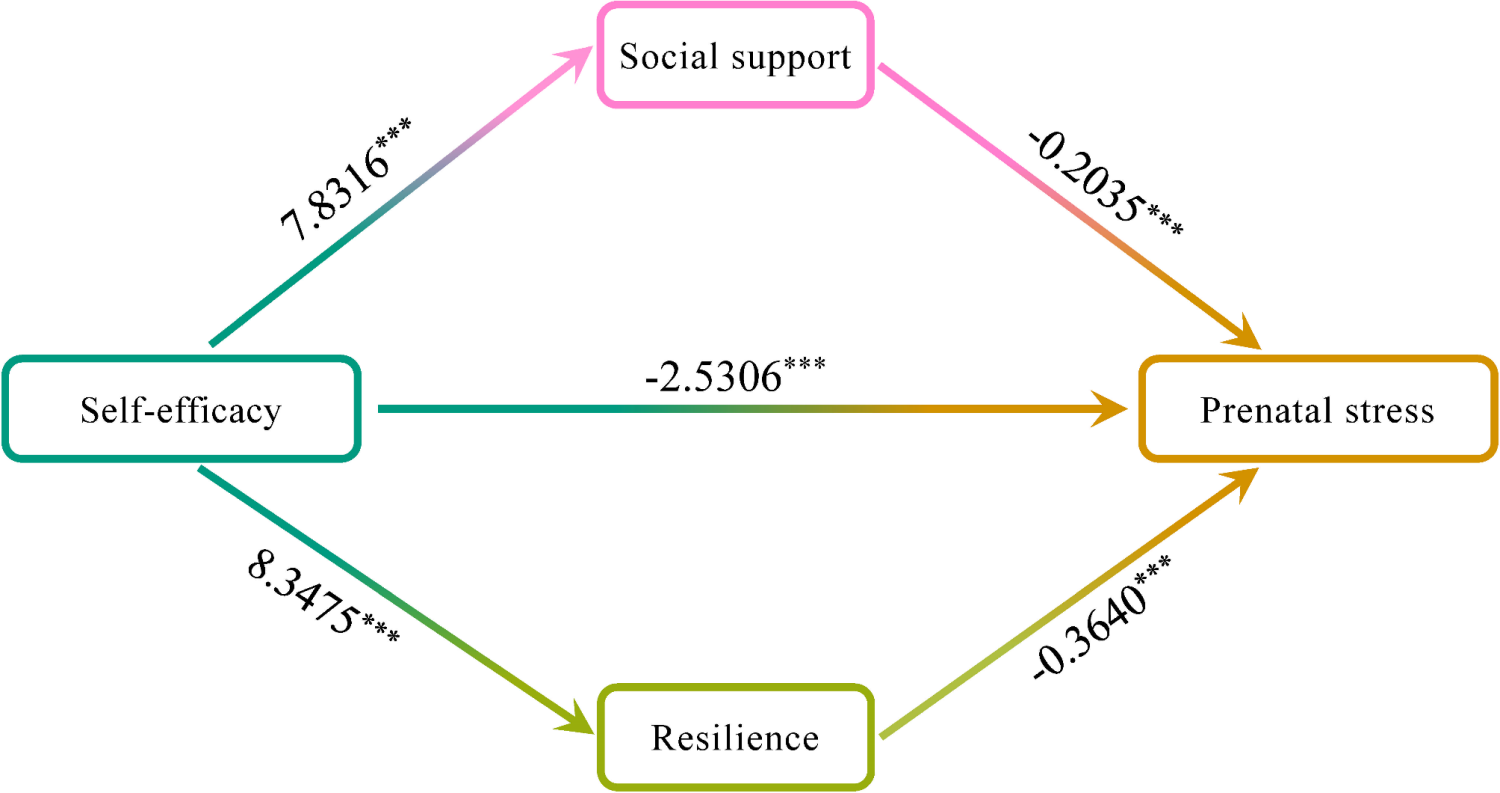
The mediating role of social support and resilience between self-efficacy and prenatal stress. *** *P* < 0.001

Subsequently, the parallel mediating effects of social support and resilience on the association between self-efficacy and prenatal stress were identified (Fig. 3). The overall mediating effect of social support and resilience was − 4.6321 (95% CI: -6.0029 to -3.3700), accounting for 64.67% of the total effect. Specifically, the mediating effect attributed to social support was − 1.5933 (95%: CI -2.2907 to -0.9496), accounting for 22.24% of the total effect, while the mediating effect related to resilience was − 3.0388 (95% CI: -4.3844 to -1.7135), accounting for 42.43% of the total effect (Table 5). The difference between the regression coefficients of the two indirect paths was tested using the bootstrap method. The upper and lower limits of the CI included 0, indicating a lack of statistical significance in the difference between the effect sizes of the two paths (*P* = 0.1415). In conclusion, social support and resilience jointly exert a partial mediating effect on the association between self-efficacy and prenatal stress, accounting for 64.67% of the total effect.

**Table 5:** The parallel mediating effect of Social Support and Resilience on the relationship between Self-efficacy and Prenatal stress (incompletely standardized indirect effect(s) of X on Y)

Model Pathways	Effect	Boot SE	95% CI		Relative Mediation
			BootLL CI	BootUL CI	Effect %
Direct effect	-2.5306	0. 7646	-4.0309	-1.0303	35.33%
self-efficacy →social support →prenatal stress	-1.5933	0.3453	-2.2907	-0.9496	22.24%
self-efficacy →resilience →prenatal stress	-3.0388	0.6832	-4.3844	-1.7135	42.43%
Total indirect effect	-4.6321	0. 6725	-6.0029	-3.3700	64.67%
Total effect	-7.1628	0.5012	-8.1462	-6.1793	100.00%

## Discussion

Social support and resilience mediated the relationship between self-efficacy and prenatal stress among pregnant women in China. This suggests that Chinese pregnant women could enhance the stress-reducing effects of self-efficacy by increasing their social support networks and resilience. It is believed that these findings will enhance our understanding of the underlying mechanisms between self-efficacy and prenatal stress and provide new evidence for effective interventional and prevention strategies to address prenatal stress in the future.

In the present study, 88.05% of pregnant women reported experiencing prenatal stress, which was comparatively higher than the 12–84% range observed in several studies [37–39]. Similar to our findings, a study conducted in Chongqing reported a prevalence of 91.86% [6]. Overall, our findings indicated that prenatal stress is a common mental health issue among Chinese perinatal women and that the prevalence of prenatal stress in the maternal population is significantly higher than that in the general adult population. Pregnancy-specific stress encompasses the anxieties, concerns, and fears experienced by expectant mothers [8]. Pregnant women experience stresses inherent to pregnancy, including relationship concerns, parental concerns, physical changes, apprehensions about labour and delivery, as well as concerns regarding the infant’s health and future care, work responsibilities, and many other possible issues [7, 40]. These unique stressors specific to pregnancy, in combination with general stressors, contribute to a heightened level of stress experienced by pregnant women compared with the general population.

The results suggest that primiparous women report higher stress levels in relation to pregnancy. This stress encompasses concerns related to physical symptoms, the health of the foetus, impending childbirth, the adjustment to their newfound maternal role, financial changes, and interpersonal relationships [41]. Primiparous women warrant special care and attention. This is due to the unique physiological and psychological changes that occur during pregnancy, coupled with the transition to the maternal role in society, which in itself could be a significant stressor. Identifying pregnant women or specific groups at risk of experiencing stress is of paramount importance, as it could help prevent adverse outcomes in maternal and perinatal healthcare [38].

Based on Bandura’s theory [42], it becomes apparent that perceived self-efficacy levels could play a vital role in regulating and triggering prenatal stresses. Our findings revealed that self-efficacy exerts direct effects on the symptoms of prenatal stress, accounting for 35.33% of the total variance. This implies that individuals with elevated self-efficacy levels possess a valuable resource for managing and mitigating prenatal stress symptoms. Furthermore, in alignment with physiological responses associated with self-efficacy [43], a positive belief in one’s ability to succeed at challenging tasks is established. Given the physiological changes experienced by pregnant women coupled with changes in social roles, those with a heightened sense of self-efficacy are better equipped to prepare for childbirth, explore new interests, embrace changes, and adapt to the various physical and psychological discomforts and environmental changes, thereby enhancing their resilience [44]. Additionally, pregnant women who possess a strong sense of self-efficacy, enabling effective and confident coping mechanisms in stressful situations might exhibit stronger capabilities to overcome stress during pregnancy, thereby increasing their resilience.

Resilience is a dynamic adaptive process wherein individuals proactively respond to adverse events and make efforts to adapt to new roles or environments. One of the important findings of our study was the role of resilience in mediating 42.43% of the effects of self-efficacy on prenatal stress. This underscores the significance of resilience and the highlights benefits of incorporating it into future intervention strategies aimed at enhancing the mental health of pregnant women. Pregnant women with higher resilience levels effectively leverage available resources to accommodate significant changes and develop effective coping strategies to contend with pregnancy-related stress [45]. Increased resilience has been associated with qualities such as spirituality, sense of humour, hope, and spiritual influences, all of which constitute essential components of resilience in reducing stress, with self-efficacy serving as the key element [46]. Individuals with increased self-efficacy exhibit more prominent attributes of resilience, rendering them better equipped to cope with stress [47]. These findings underscore the significance of resilience and the benefits of incorporating it into future intervention strategies designed to improve mental health in pregnant women.

Another important finding of our study was that social support mediated 22.24% of the effect of self-efficacy on prenatal stress. This underscores the particular significance of social support during pregnancy, a period marked by numerous changes and stressful events, including physical and psychological changes, role changes, concerns related to medical issues, both for themselves and their child, as well as medical appointments, among others. Social support is recognised as a valuable buffer against stress, and it is postulated that individuals can optimise their social support networks by enhancing their self-efficacy [48]. Increasing self-efficacy among pregnant women is linked to enhanced access to healthcare services and the availability of resources to address pregnancy-related concerns, thereby facilitating the acquisition of emotional support and effective coping strategies for managing general or specific stresses associated with pregnancy. This, in turn, contributes to the enhanced mental well-being of pregnant women. Therefore, prenatal healthcare providers should pay more attention to women with self-reported low social support and promptly provide mental support to address prenatal stress.

To the best of our knowledge, this is the first study to evaluate the relationship between self-efficacy and prenatal stress mediated by social support and resilience among pregnant women. Our findings offer a fresh perspective on how self-efficacy affects prenatal stress, effectively bridging a critical research gap. Additionally, they open up new horizons for research in the field of prenatal stress and provide a scientific basis for addressing prenatal stress through the mediating effect of social support and resilience. While it is essential to conduct further longitudinal studies to validate our findings, they can be used to develop interventions to prevent symptoms of prenatal stress, improve prenatal social support, and enhance prenatal mental resilience. Potential interventions include the organisation of support groups, the implementation of mindfulness-based courses, and the introduction of stress management programs. Moreover, fostering self-efficacy in pregnant women through positive family and workplace interactions can prove beneficial in preventing prenatal stress. Furthermore, recognising the significant impact of social support and resilience on prenatal stress, social support counselling and mental resilience modelling should be considered to help pregnant women effectively address prenatal stress-related issues before their occurrence. Thus, our findings hold practical significance in safeguarding the mental well-being of Chinese pregnant women.

This study has several limitations. First, while parallel mediation analysis represents a valuable approach by allowing the examination of multiple mediators within a single model to estimate the direct and indirect effects of the variables simultaneously, it is important to note that this analysis might not fully capture the complexity of the relationships involved. Further studies employing other analyses to comprehensively explore and validate these complexities are warranted. Second, many factors influence prenatal stress, and it is possible that some of these factors could also serve as mediators in the relationship between self-efficacy and prenatal stress. Third, the use of a convenience sampling method, while advantageous for expedited data collection and wide survey distribution, introduces the potential for selection bias. Consequently, the generalisability of the findings might be limited. Lastly, the study’s cross-sectional design, conducted exclusively in three hospitals in China, limits the generalisability of the findings among pregnant women belonging to other populations.

## Conclusion

The findings of this study revealed that the prevalence of prenatal stress in China was high (88.05%). Moreover, this study demonstrated that social support and resilience mediated the relationship between self-efficacy and prenatal stress among Chinese pregnant women in their third trimester. Therefore, improving social support, resilience, and self-efficacy among pregnant women holds promise as a means to prevent and alleviate prenatal stress.

## Data Availability

The raw data of the current study would be available from the corresponding author on reasonable request.

## References

[1] Guardino CM, Schetter CD (2014). Coping during pregnancy: a systematic review and recommendations. Health Psychol Rev.

[2] Alzboon G, Vural G. Factors influencing the quality of life of healthy pregnant women in North Jordan. Med (Kaunas) 2019, 55(6). 10.3390/medicina55060278 PMC6631935 31208100

[3] Rodrigues OM, Schiavo Rde A (2011). [Stress in pregnancy and puerperium: a correlation with postpartum depression]. Rev Bras Ginecol Obstet.

[4] Bennett AE, Kearney JM. Factors Associated with maternal wellbeing at four months Post-partum in Ireland. Nutrients 2018, 10(5). 10.3390/nu10050609 PMC5986489 29757937

[5] Carolan-Olah M, Barry M (2014). Antenatal stress: an Irish case study. Midwifery.

[6] Tang X, Lu Z, Hu D, Zhong X (2019). Influencing factors for prenatal stress, anxiety and depression in early pregnancy among women in Chongqing, China. J Affect Disord.

[7] Herbell KPD, Zauszniewski Ja PhD R-BF (2019). Stress Experiences and Mental Health of pregnant women: the mediating role of Social Support. Issues Ment Health Nurs.

[8] Lobel M, Cannella DL, Graham JE, DeVincent C, Schneider J, Meyer BA (2008). Pregnancy-specific stress, prenatal health behaviors, and birth outcomes. Health Psychol.

[9] Faramarzi M, Kheirkhah F, Barat S, Cuijpers P, O’Connor E, Ghadimi R, Hajian-Tilaki K, Pahlavan Z, Hamidia A, Mirtabar SM (2020). Prevalence and factors related to psychiatric symptoms in low risk pregnancy. Casp J Intern Med.

[10] Haghparast E, Faramarzi M, Hassanzadeh R (2016). Psychiatric symptoms and pregnancy distress in subsequent pregnancy after spontaneous abortion history. Pak J Med Sci.

[11] Koolhaas JM, Bartolomucci A, Buwalda B, de Boer SF, Flugge G, Korte SM, Meerlo P, Murison R, Olivier B, Palanza P (2011). Stress revisited: a critical evaluation of the stress concept. Neurosci Biobehav Rev.

[12] Moss KM, Simcock G, Cobham V, Kildea S, Elgbeili G, Laplante DP, King S (2017). A potential psychological mechanism linking disaster-related prenatal maternal stress with child cognitive and motor development at 16 months: the QF2011 Queensland Flood Study. Dev Psychol.

[13] Vesga-Lopez O, Blanco C, Keyes K, Olfson M, Grant BF, Hasin DS (2008). Psychiatric disorders in pregnant and postpartum women in the United States. Arch Gen Psychiatry.

[14] Yu ZM, Van Blyderveen S, Schmidt L, Lu CH, Vanstone M, Biringer A, Sword W, Beyene J, McDonald SD (2023). Do psychological and behavioural factors change over pregnancy?. J Obstet Gynaecol Can.

[15] Scheyer K, Urizar GG (2016). Altered stress patterns and increased risk for postpartum depression among low-income pregnant women. Arch Womens Ment Health.

[16] Hsiung Y, Lee CF, Chi LK, Huang JP. Moving for my baby! Motivators and perceived barriers to facilitate readiness for physical activity during pregnancy among obese and overweight women of Urban Areas in Northern Taiwan. Int J Environ Res Public Health 2021, 18(10). 10.3390/ijerph18105275 PMC8156013 34063538

[17] Ngai FW, Chan SW (2012). Stress, maternal role competence, and satisfaction among Chinese women in the perinatal period. Res Nurs Health.

[18] Tsai YJ, Hsu YY, Hou TW, Chang CH (2018). Effects of a web-based Antenatal Care System on maternal stress and self-efficacy during pregnancy: a study in Taiwan. J Midwifery Womens Health.

[19] Behmard V, Bahri N, Mohammadzadeh F, Noghabi AD, Bahri N (2022). Relationships between anxiety induced by COVID-19 and perceived social support among Iranian pregnant women. J Psychosom Obstet Gynaecol.

[20] Gilbert L, Gross J, Lanzi S, Quansah DY, Puder J, Horsch A (2019). How diet, physical activity and psychosocial well-being interact in women with gestational Diabetes Mellitus: an integrative review. BMC Pregnancy Childbirth.

[21] Pasha H, Faramarzi M, Chehrazi M, Esfandyari M, Shafierizi S (2021). Role of social capital and self-efficacy as determinants of stress in pregnancy. Tzu Chi Med J.

[22] Salomonsson B, Gullberg MT, Alehagen S, Wijma K (2013). Self-efficacy beliefs and fear of Childbirth in nulliparous women. J Psychosom Obstet Gynaecol.

[23] Jin X, Xu X, Qiu J, Xu Z, Sun L, Wang Z, Shan L (2021). Psychological resilience of second-pregnancy women in China: a cross-sectional study of influencing factors. Asian Nurs Res (Korean Soc Nurs Sci).

[24] Garcia-Leon MA, Caparros-Gonzalez RA, Romero-Gonzalez B, Gonzalez-Perez R, Peralta-Ramirez I (2019). Resilience as a protective factor in pregnancy and puerperium: its relationship with the psychological state, and with hair cortisol concentrations. Midwifery.

[25] Van Haeken S, Braeken M, Nuyts T, Franck E, Timmermans O, Bogaerts A (2020). Perinatal resilience for the First 1,000 days of life. Concept Analysis and Delphi Survey. Front Psychol.

[26] Ma R, Yang F, Zhang L, Sznajder KK, Zou C, Jia Y, Cui C, Zhang W, Zhang W, Zou N (2021). Resilience mediates the effect of self-efficacy on symptoms of prenatal anxiety among pregnant women: a nationwide smartphone cross-sectional study in China. BMC Pregnancy Childbirth.

[27] Tuxunjiang X, Wumaier G, Zhang W, Sailike B, Wang X, Jiang T (2022). The relationship between positive psychological qualities and prenatal negative emotion in pregnant women: a path analysis. Front Psychol.

[28] Chen CH, Chen HM, Huang TH (1989). Stressors associated with pregnancy as perceived by pregnant women during three trimesters. Gaoxiong Yi Xue Ke Xue Za Zhi.

[29] Zhou X, Liu H, Li X, Zhang S (2021). Fear of Childbirth and Associated Risk factors in healthy pregnant women in Northwest of China: a cross-sectional study. Psychol Res Behav Manag.

[30] Zimet GD, Powell SS, Farley GK, Werkman S, Berkoff KA (1990). Psychometric characteristics of the Multidimensional Scale of Perceived Social Support. J Pers Assess.

[31] Zhou K, Li H, Wei X, Li X, Zhuang G (2017). Relationships between perceived social support and retention among patients in Methadone maintenance treatment in mainland China. Psychol Health Med.

[32] Connor KM, Davidson JR (2003). Development of a new resilience scale: the Connor-Davidson Resilience Scale (CD-RISC). Depress Anxiety.

[33] Cheng C, Dong D, He J, Zhong X, Yao S (2020). Psychometric properties of the 10-item Connor-Davidson Resilience Scale (CD-RISC-10) in Chinese undergraduates and depressive patients. J Affect Disord.

[34] Huang J, Xu L, Xu Z, Luo Y, Liao B, Li Y, Shi Y (2022). The relationship among pregnancy-related anxiety, perceived social support, family function and resilience in Chinese pregnant women: a structural equation modeling analysis. BMC Womens Health.

[35] Schwarzer R, Renner B (2000). Social-cognitive predictors of health behavior: action self-efficacy and coping self-efficacy. Health Psychol.

[36] Cheung SK, Sun SY (1999). Assessment of optimistic self-beliefs: further validation of the Chinese version of the General Self-Efficacy Scale. Psychol Rep.

[37] Kingston D, Heaman M, Fell D, Dzakpasu S, Chalmers B (2012). Factors associated with perceived stress and stressful life events in pregnant women: findings from the Canadian maternity experiences Survey. Matern Child Health J.

[38] Woods SM, Melville JL, Guo Y, Fan MY, Gavin A (2010). Psychosocial stress during pregnancy. Am J Obstet Gynecol.

[39] Dolatian M, Sharifi N, Mahmoodi Z, Fathnezhad-Kazemi A, Bahrami-Vazir E, Rashidian T (2020). Weight gain during pregnancy and its associated factors: a path analysis. Nurs Open.

[40] Levine TA, Grunau RE, Segurado R, Daly S, Geary MP, Kennelly MM, O’Donoghue K, Hunter A, Morrison JJ, Burke G (2017). Pregnancy-specific stress, fetoplacental haemodynamics, and neonatal outcomes in women with small for gestational age pregnancies: a secondary analysis of the multicentre Prospective Observational Trial to Optimise Paediatric Health in Intrauterine Growth Restriction. BMJ Open.

[41] Dahlen HG, Barclay LM, Homer CS (2010). The novice birthing: theorising first-time mothers’ experiences of birth at home and in hospital in Australia. Midwifery.

[42] Smoktunowicz E, Cieslak R, Demerouti E (2017). Interrole conflict and self-efficacy to manage work and family demands mediate the relationships of job and family demands with stress in the job and family domains. Anxiety Stress Coping.

[43] Brandao S, Mendonca D, Dias CC, Pinto TM, Dennis CL, Figueiredo B (2018). The breastfeeding self-efficacy scale-short form: psychometric characteristics in Portuguese pregnant women. Midwifery.

[44] Khresheh RM, Ahmad NM (2018). Breastfeeding self efficacy among pregnant women in Saudi Arabia. Saudi Med J.

[45] Howell KH, Miller-Graff LE, Schaefer LM, Scrafford KE (2020). Relational resilience as a potential mediator between adverse childhood experiences and prenatal depression. J Health Psychol.

[46] Schaefer LM, Howell KH, Sheddan HC, Napier TR, Shoemaker HL, Miller-Graff LE (2021). The Road to Resilience: strength and coping among pregnant women exposed to intimate Partner Violence. J Interpers Violence.

[47] Roberto A, Sellon A, Cherry ST, Hunter-Jones J, Winslow H (2020). Impact of spirituality on resilience and coping during the COVID-19 crisis: a mixed-method approach investigating the impact on women. Health Care Women Int.

[48] Ernsting A, Knoll N, Schneider M, Schwarzer R (2015). The enabling effect of social support on vaccination uptake via self-efficacy and planning. Psychol Health Med.

